# Discussions upon Surgery

**Published:** 1894-02

**Authors:** 


### DISCUSSIONS UPON SURGERY.

[Proceedings of I. H. A.]

### Dr. H. C. Allen's Paper, "The Surgical Value of Silicea."

Dr. Holmes—I have for many years been a careful student of the Germ Theory, and the more I study it and scientific medicine, the better homœopath I become. We are taught from the Germ Theory, that there can be no pus without infection from pus microbes, and yet Silicea started up suppuration in this case without any infection in an internal cavity. There is a question in my mind whether the position of the ball was certainly made out. It is hard to understand why the ball traveled so far out of its direct path, if the location of the ball was correctly diagnosed.

Dr. H. C. Allen—I examined the man about ten years ago, and I made out the position of the ball as plainly as anything could be. Last fall I located the ball in the same position and by the same means. Dr. Boynton, our surgeon, also made an examination and came to the same conclusion.

Dr. Fowler—I remember this case distinctly and am much gratified to hear this final report, for I had heard in some way that the patient had died, a few days after reaching home. I am glad to hear that he recovered and also that I was correct in my judgment, for I told him that if there was a foreign body in him it would be found at lower part of right lung or on upper surface of liver. The dullness on percussion could be distinctly marked out.

Dr. A. R. Morgan—It is remarkable that that sinus could have remained there so many years without discharge. Perhaps the pus discharged upward into the lungs. I do not think there could have been closure, it must have remained open and the Silicea started the process of expulsion.

Dr. H. C. Allen—The point of exit was six inches below the point of entrance.

Dr. Fowler—Silicea is the enemy of foreign bodies; it works them out. I have seen it do it many times in the case of carious and dead teeth.

Dr. Hanchett—Was the Silicea selected empirically or according to indications.

Dr. H. C. Allen—According to the symptoms. I hesitated a year ago to give, because if you want trouble give Silicea in cases of foreign bodies in the tissues.

---

### Dr. A. R. Morgan's Paper, "Intussusception Cured without the Knife."

Dr. H. C. Allen—If a fatal case will add to the interest of the discussion I have such a case to report. The lady was taken sick at four P. M. The next morning she died in spite of all efforts to save her. The remedies did not stop for five minutes the progress of the disease. The *post-mortem* showed the difficulty to be intussusception.

Dr. W. L. Reed—One of our students in St. Louis had such a case soon after graduating. He came to see me two or three times about it, and finally sent for me. She had a cold sweat all over, a pinched nose, and a state of great prostration. We gave her a remedy, I think it was Arsenicum. After this she seemed to get better and rested tolerably well that night. I did not go again until sent for, several days after. I found her in *articulo mortis*.

Dr. Holmes—I reported a case of this kind in *The Clinique* about 1885, about which the members of the Society and the Faculty of the Hahnemann College were very kind in praising and commenting on it. The symptoms were very grave, and the good feature about it was that I managed to save my patient. He was a boy seven or eight years old, who for two weeks during his sickness had not had a passage of the bowels. There was great tenesmus, and toward the last fecal vomiting. The vomited matter was almost identical with a watery diarrhœa of a horrible odor, and, as the boy said—taste. Abdomen was tympanitic; face pale and collapsed. For treatment there were many remedies used single and in alternation (remember this was some years ago). I used galvanism, putting one electrode in the anus and the other in the mouth, flattering myself that the current might take the line of the intestine and disentangle the obstruction. At the same time I gave Belladonna and within one hour there was a passage of the bowels, and the little fellow recovered and is well to-day. What did it I do not know; whether it was the Belladonna or the galvanism is a question that cannot now be answered.

---

### Dr. E. Adams' Paper, "Cases Cured by the Remedy."

Dr. J. H. Lewis—Belladonna, which did such good work in this case, was not only indicated by the symptoms but also an antidote to the Opium, which had been used and had poisoned the case.

---

### Dr. E. E. Case's Paper, "Surgical Cases Cured by the Indicated Remedy, Cataract, Goitre, and Polypus Nasi."

Dr. Taft—I should like to ask Dr. Case if he is always so successful in such cases of polypus.

Dr. E. E. Case—I have never treated but two nasal polypi and in both of them Calcarea-carb. was indicated and in both cured them.

Dr. Peason—Last winter I was calling at the house of a friend. One of the guests complained of a severe coryza of a chronic character. She had been unable to get relief from any physician she had ever tried. It was such a pretty case for Calc-carb., without regard to the polypus, that I told the lady I was so confident that there was a remedy that would not only relieve the coryza, but also make her a sound woman that I would treat her for nothing. She consented and I examined the nose. I found a polypus occupying the greater part of the left side of the nasal fossa. She received one dose of Calc-carb.$^{10m}$. In one week she returned saying there was no perceptible improvement. Still I found she was stronger, could sleep better, and there was less exhaustion. In another week she came back reporting no improvement in nose, but general condition perceptibly better. I then gave her a dose of the same 55M, and put her upon Placebo. She went into the country and I did not see her for three weeks. After she had been there about two weeks as a result of some exercise a mass came from her nose followed by hemorrhage. She found it to be a fleshy mass. After that no coryza, and her general health has been almost a miracle to herself and friends.

Dr. Hanchett—One case that came to my notice not long before I left home, to come here, was so peculiar that I should like to report it. It was a case similar to the last one reported by Dr. Case. There were no polypi, but there was follicular pharyngitis, with cheesy concretions, forming in throat, and great susceptibility to drafts. He would get stuffed up at night so that there was difficulty in breathing. There was also constipation for which he was using Hall's method of flushing the colon. The indications were clear for Nux-vomica. I gave him the 200th and Placebo; after four weeks I examined his throat, and found the tonsils normal.

This patient had come to me because the specialists had wanted to sear them. He said that for a week after beginning my treatment he had spit more cheesy lumps than ever before, and that the medicine had acted on his bowels. What I wanted to inquire about was whether this rapid exfoliation of cheesy lumps could properly be set down as due to the remedy.

Dr. Kennedy—Certainly, that was simply a homœopathic aggravation.

Dr. H. C. Allen—In Dr. Pearson's case we have a sample of what we are prone to forget, and that is that our best cures are made when we let a single dose alone. In Dr. Hanchett's case the aggravation was assuredly due to the remedy.

Our greatest trouble has been to keep from giving medicine unnecessarily. I write down the symptoms of all cases of a chronic character, and when the patient comes in again, saying they are no better, I take down my record book and inquire about the different symptoms given at the first visit, and if some or any of the symptoms are better, it is an indication to let the patient alone. No especial attention need be paid to the polypus, the cheesy secretion, or the hemorrhoidal tumors. The way to keep yourself from repeating unnecessarily is to put down in black and white the symptoms, and at the succeeding visits go over them carefully one by one. It is the only scientific way of finding out just when a second dose of the same or another medicine is needed. This applies to all chronic cases irrespective of potency.

Dr. A. R. Morgan—There is one thing that puzzles me very much, and that is, how a broken dose differs from repetition. I can't see any difference. In many of my cases I have been in the habit of giving medicine in two or three successive doses, for we all get into habits or ruts, but I do not call it one dose. The term, broken doses, seems to me to be only a subterfuge or a way of excusing bad practice. If you do it, call it what it is, repetition.

Dr. H. C. Allen—I do not always give the so-called broken dose, I sometimes do it in acute cases, when I cannot stay long enough to see the case through. I think the rule as given in *The Organon* is to give doses enough to make an impression and then wait until that impression dies out, or until the improvement stops. If I were absolutely sure every time and in every case that the single dose on the tongue was the best means of curing I would always do it, of course.

Dr. A. R. Morgan—Some of the best cures I have ever made, have been with a single dose.

---

### Dr. F. O. Pease's Paper, "Simillimum in Surgery."

### Dr. W. L. Reed's Paper, "Two Cases of Cancer of the Mammæ Cured by the Internal Remedy."

Dr. A. Fisher—These cases remind me of one which came under my care some years ago. It was a case of tumor of the breast, and I treated it as well as I knew how at that time, but

I was unsuccessful. A professor of surgery in an allopathic college said it was a case of fungus hæmatodes, and advised immediate operation, which was assented to. It was successful as far as the breast went, but only a short time after tubercular symptoms developed and the patient died. The *post-mortem* revealed tuberculosis, the lungs being full of melænœ. I held at the time and since that the external disease was simply driven in by the operation.

Dr. Clark—I have a case or two similar to those reported. A diagnosis of cancer had been made by a professor of surgery in New York. There could be no doubt of the cancerous condition, they said.

I gave it as my opinion that it was not cancerous. The remedy that benefited her the most was Pulsatilla. At first I gave her one dose of Arnica, and then Puls., with great benefit. The involved glands softened and grew smaller. One symptom that came up prominently was itching of the nipple, which one dose of Agaricus removed, together with the whole trouble, after a time. There was an erectile condition of the nipple at the time. She is now well. One physician, who examined her, did not advise immediate removal, but told her to wait, until a diagnosis could be made with certainty.

Bureau closed.

